# Light‐Modulated Humidity Sensing in Spiropyran Functionalized MoS_2_ Transistors

**DOI:** 10.1002/smll.202404633

**Published:** 2024-09-12

**Authors:** Adrián Tamayo, Wojciech Danowski, Bin Han, Yeonsu Jeong, Paolo Samorì

**Affiliations:** ^1^ Institut de Science et d'Ingénierie Supramoléculaires Université de Strasbourg & CNRS 8 Allée Gaspard Monge Strasbourg 67000 France; ^2^ Present address: Faculty of Chemistry University of Warsaw Warsaw 02‐093 Poland

**Keywords:** 2D semiconductors, humidity sensors, multistimuli‐responsive, photochromic, photodetector, spiropyran

## Abstract

The optically tuneable nature of hybrid organic/inorganic heterostructures tailored by interfacing photochromic molecules with 2D semiconductors (2DSs) can be exploited to endow multi‐responsiveness to the exceptional physical properties of 2DSs. In this study, a spiropyran‐molybdenum disulfide (MoS_2_) light‐switchable bi‐functional field‐effect transistor is realized. The spiropyran‐merocyanine reversible photo‐isomerization has been employed to remotely control both the electron transport and wettability of the hybrid structure. This manipulation is instrumental for tuning the sensitivity in humidity sensing. The hybrid organic/inorganic heterostructure is subjected to humidity testing, demonstrating its ability to accurately monitor relative humidity (RH) across a range of 10%–75%. The electrical output shows good sensitivity of 1.0% · (%) RH^−1^. The light‐controlled modulation of the sensitivity in chemical sensors can significantly improve their selectivity, versatility, and overall performance in chemical sensing.

## Graphical abstract

A hybrid material combining molybdenum disulfide (MoS_2_) and spiropyran for humidity and light responsiveness is developed. Ultraviolete (UV) irradiation tunes the wettability of MoS_2_ decorated by spiropyran, enhancing the humidity sensitivity. Water adsorption and photochromic doping significantly alter the optoelectronic properties of MoS_2_ flakes, greatly improving device performance.

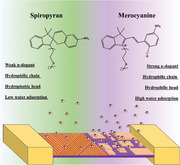

## 1 Introduction

The atomic thickness and the exceptional charge transport characteristics of 2D semiconductors (2DSs) render their electronic properties highly sensitive to environmental changes. As a result 2DSs are ideal platforms for development of high‐performance devices for chemical sensing applications^[^ ^1^ ^]^ and neuromorphic devices due to their multi‐parametric electrical response which can be of paramount importance in the context of post‐Moore electronic technologies.^[^ ^2^, ^3^ ^]^ 2DSs are capable of responding to a large portfolio of external stimuli such as radiation,^[^ ^4^, ^5^, ^6^, ^7^, ^8^, ^9^ ^]^ humidity,^[^ ^10^, ^11^, ^12^, ^13^ ^]^ temperature,^[^ ^14^, ^15^, ^16^ ^]^ pressure,^[^ ^17^ ^]^ or the presence of pollutants.^[^ ^18^, ^19^, ^20^, ^21^, ^22^ ^]^ However, the development of highly‐sensitive and high‐performance chemical sensors based on pristine 2DSs suffers from significant reliability issues associated with poor selectivity, low reversibility, and environmental instability.^[^ ^23^, ^24^, ^25^, ^26^, ^27^ ^]^

The adsorption of small functional molecules onto the surface of 2DSs allows to impart them specific new functions or enhance their inherent unique characteristics.^[^ ^28^, ^29^, ^30^, ^31^, ^32^ ^]^ In particular, fabrication of the hybrid organic/2DSs van der Waals heterostructures has been shown to constitute an efficient method to achieve controlled doping or to endow 2DSs with stimuli‐responsiveness to specific external inputs.^[^ ^33^, ^34^, ^35^, ^36^ ^]^ The widest portfolio of molecular functionalities that can be programmed via chemical synthesis provides tools for the decoration of 2DSs with receptors capable to specifically interact with the analyte of choice, thereby conferring high selectivity to the sensing event. Inspired by the 2D‐2D van der Waals heterostructures, the generation of ultrathin and highly ordered molecular layers onto the basal plane of 2DSs enables the construction of hybrid organic–inorganic structures, thereby enhancing the functional complexity of materials. By mastering this strategy, using molecular switches as reconfigurable dopants, hybrid materials and electronic devices thereof capable of responding to a wide range of external inputs have been developed. This is particularly interesting for application in chemical sensing through the fabrication of devices possessing on‐demand selectivity and sensitivity, upon using molecular switches whose different states exhibit a diverse affinity for the analyte of choice.^[^ ^37^, ^38^, ^39^, ^40^, ^41^, ^42^ ^]^

**Figure 1:**
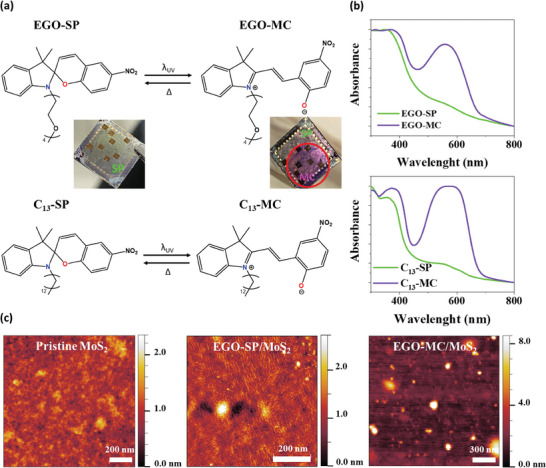
(a) Molecular structures of EGO‐SP and C_13_‐SP along the schematic representation of their isomerization. (b) UV–vis absorption spectra of EGO‐SP and C_13_‐SP films spin‐coated on quartz substrates. (c) AFM topography images of pristine MoS_2_, EGO‐SP/MoS_2,_ and EGO‐MC/MoS_2_ supported on BCB/SiOx.

The electronic characteristics of 2DSs can be modulated by interfacing them with organic photochromic molecules, such as diarylethene,^[^ ^43^, ^44^ ^]^ azobenzene,^[^ ^38^, ^45^, ^46^, ^47^, ^48^ ^]^ or spiropyran.^[^ ^41^, ^49^, ^50^ ^]^ Photochromic molecules can be reversibly toggled between at least two isomers when exposed to light at specific wavelengths in a process characterized by a high sensitivity to a narrow range of wavelengths, a fast response time and excellent reversibility. Among photochromic molecules, spiropyran (SP) undergoes reversible photochemical isomerization between a neutral form (SP) and a zwitterionic merocyanine (MC) form. The photo‐isomerization of the neutral SP with ultraviolet (UV) light (λ = 365 nm) yielding the zwitterionic MC form is accompanied by a major enhancement in the intrinsic dipole moment (**Figure** **1**a).^[^ ^51^ ^]^ It can be reconverted to SP either thermally or via irradiation with visible light (560 nm).^[^ ^51^, ^52^ ^]^ The reversible SP‐to‐MC isomerization in heterostructures based SP/2DSs has been used to control the charge transport through the 2DS.^[^ ^41^, ^49^, ^50^ ^]^ The interfacing of the molecules with the 2DSs can be further adjusted through the chemical functionalization of the SP core with side‐chains, to achieve an enhanced control over the molecular self‐assembly behavior at surfaces and interfaces. Such functionalization also influences the interaction with the environment through specific non‐covalent forces like electrostatic ones. In particular, the functionalization with organic moieties containing heteroatoms such as oxygen groups (hydroxyl, carboxyl, vinyl alcohol, ethylene glycol units, etc) can promote dipole–dipole interactions with water molecules in the atmosphere ^[^ ^53^, ^54^, ^55^ ^]^ while playing a key role in the molecular self‐assembly.^[^ ^56^, ^57^, ^58^ ^]^ Owing to these characteristics, the dressing of 2DS surfaces with functionalized spiropyran molecules represents a useful approach to confer them a reversible light‐controlled wettability.^[^ ^59^, ^60^ ^]^

In this paper, we demonstrate light‐tuneable humidity sensing based on SP derivative/2DSs hybrid structures. We have focused our attention on 2D molybdenum disulfide (MoS_2_) in its 2H phase as prototypical n‐type semiconductor possessing excellent mechanical and electrical properties that worsen upon adsorption of water molecules on its surface.^[^ ^61^, ^62^ ^]^ We show that the decoration of mechanically exfoliated MoS_2_ with solution‐processed spiropyrans functionalized with either ethylene glycol chains (EGO‐SP) or alkyl chains (C_13_‐SP) (Figure 1a) is a viable strategy to tune the field‐effect transistors (FETs) sensitivity to humidity changes by exploiting a subtle yet non‐dynamic control over the interaction with the water molecules in the environment. Ethylene glycol chains, in particular, are often employed as functional groups to enhance the hygroscopic properties of materials.^[^ ^13^, ^63^, ^64^ ^]^ In addition to the role of the side chains grafted onto the spiropyran core, the reversible photo‐isomerization between the neutral spiropyran and the zwitterionic merocyanine was revealed to determine a major and dynamic change in the hydrophilicity of the hybrid structure, yielding a tuneable, that is, dynamic, sensitivity in humidity sensing. Our responsive MoS_2_ FETs displayed a light‐modulated increase in electron transport as evidenced by an enhancement in current exceeding 50% upon EGO‐SP to EGO‐MC photo‐isomerization, yielding a sensitivity in humidity detection up to 1.0% · (%) RH^−1^.

## 2 Results and Discussion

1 mg·mL^‐1^ solutions of EGO‐SP and C_13_‐SP in toluene were spin‐coated onto the chosen solid substrate, followed by a mild thermal annealing at 80 °C for 10 min, in order to trigger the complete thermalization to the spiropyran form and achieve the full evaporation of solvent molecules. The responsiveness of SP isomers supported onto quartz substrates to UV light exposure and thermal treatment was assessed spectroscopically. The UV–vis spectra of both systems, that is, EGO‐SP (Figure 1b, **top**) and C_13_‐SP (Figure 1b, **bottom**) evidence the existence of the ring‐closed spiropyran isomer, before the UV irradiation, and the ring‐open zwitterionic merocyanine form, after the UV irradiation. Making use of exposure to green light or thermal annealing at 80 °C, the back‐isomerization was accomplished yielding the SP isomer, as evidenced by its characteristic band at 365 nm. Upon irradiation with UV light, an intense band emerges at 530 nm, being the hallmark of the MC isomer (violet).^[^ ^65^ ^]^ Thermal treatment (at 80 °C) yielded an almost complete recovery of the initial SP spectrum. These data provide evidence for the facile and reversible isomerization of the spiropyran derivatives taking place in spin‐coated films.

The changes in absorption at 550 nm during the EGO‐SP to MC photo‐switching and the subsequent relaxation on quartz substrate were monitored with UV–vis spectroscopy (Figure S1a,b, Supporting Information). The typical band of MC chromophores (550 nm) appeared after a few seconds of UV (365 nm) irradiation. The absorption at 550 nm continued to increase under UV irradiation until reaching a full photo‐conversion after ca. 180–240 s of irradiation, which can be fitted with a simple monoexponential equation indicating first‐order kinetics (Figure S1c, Supporting Information).

The MC isomer can revert to SP isomer through thermal treatment, or over time as evidenced by a decrease in absorbance at 550 nm. At room temperature, the photo‐conversion from the MC to SP form occurred after more than 4 h indicating sufficient thermal stability of MC form for sensing experiments (see Figure S1a, Supporting Information). This process can be accelerated by thermal treatment at ca. 80 °C, with a fast photo‐conversion occurring in less than 2 min (Figure S1d, Supporting Information).

In order to study the photo‐conversion from the EGO‐SP isomer to the EGO‐MC, X‐ray photoelectron spectroscopy (XPS) was carried out on films on quartz. Figure S2 (Supporting Information) shows the binding energy of the N1s core level, which provides unambiguous identification of the isomer on the surface. The SP showed two characteristics N1s peaks, at 405.2 and 398.6 eV, corresponding to NO_2_ and N─C, respectively. Following UV irradiation, a new peak appeared at 399.6 eV, which can be ascribed to the N═C moiety of the MC isomer. Upon thermal treatment, an almost complete recovery of the initial SP spectrum was observed. These data provide evidence that photo‐isomerization takes place.

To minimize any interference of the interaction between water molecules and the hydrophilic SiO_x_ surface, the latter was passivated with 120 nm of a hydrophobic layer of divinyltetramethyl‐disiloxanebis(benzocyclobutene) (BCB). The BCB is widely used to provide a high‐quality hydroxyl‐free interface that acting as electron traps at the 2D/dielectric interface, thereby enhancing the photoresponse and reducing substrate wettability.^[^ ^38^, ^66^ ^]^ The SP molecules were spun onto SiO_x_ or BCB‐coated SiO_x_ substrates, and wettability changes upon irradiation with UV light were recorded. Figure S3 (Supporting Information) shows that the isomerization from EGO‐SP to EGO‐MC is accompanied by a decrease in the measured contact angle (CA) by 12±2°and 11±1°, for films supported on SiO_x_ and BCB/SiO_x_, respectively. Conversely, the C_13_‐SP to C_13_‐MC isomerization was characterized by a smaller reduction in the CA, amounting 5±2°and 6±1°, on SiO_x_ and BCB/SiO_x_, respectively. Hence, the larger changes in wettability are observed when the spiropyran is functionalized with ethylene glycol chains.^[^ ^59^ ^]^

The influence of ethylene glycol and alkyl side‐chain on the molecular self‐assembly on 2DS surfaces was explored by Atomic Force Microscopy (AFM) by comparing the morphologies of the bare MoS_2_ and SP molecules/MoS_2_ hybrids. Figure 1c reveals that the surface of bare MoS_2_, with a thickness between 3 and 7 nm (Figure S4, Supporting Information), is extremely smooth as evidenced by its root‐mean square roughness (R_RMS_) of 0.24±0.04 nm, as estimated on an image of 1 × 1 µm^2^. Conversely, the EGO‐SP/MoS_2_ (Figure 1c) and C_13_‐SP/MoS_2_ (Figure S5a,b, Supporting Information) surfaces displayed completely different morphologies. In particular, EGO‐SP/MoS_2_ films exhibit an increased roughness, amounting to R_RMS_ = 0.5±0.1 nm, and a lamellar morphology comprising tubular structures, with a width of 14.4±0.8 nm and a thickness of 0.4±0.2 nm, tightly packed onto the MoS_2_ surface forming oriented domains with preferential direction along the hexagonal lattices of the MoS_2_ (Figure S6, Supporting Information). On the other hand, the C_13_‐SP/MoS_2_ formed large ill‐defined aggregates on top of the MoS_2_ (Figure S5a, Supporting Information) with remarkably increased roughness (R_RMS_ = 1.3±0.8 nm). The thicknesses of the films were estimated from topographical AFM profiles in areas in which the SP layers have been scratched from the SiO_x_ close to the MoS_2_ flakes. They were found to amount to 2.7±0.3 nm and 8.8±0.9 nm for EGO‐SP and C_13_‐SP films, respectively (Figure S7, Supporting Information).

The surfaces of both samples have undergone remarkable modification when exposed to UV light. The lamellar morphology of EGO‐SP has transformed into large amorphous aggregates of EGO‐MC (Figure 1c), with an enhanced surface roughness (R_RMS_ = 1.1±0.5 nm). Similarly, the spatially extended crystals observed in C_13_‐SP films rearranged into large amorphous drops of C_13_‐MC (Figure S5c, Supporting Information) with a smaller roughness (R_RMS_ = 4.1±0.9 nm). Upon thermal annealing at 80 °C of the EGO‐MC, the film got smoother again, as evidenced by a roughness of (0.6±0.3 nm), and the lamellar structure typical of EGO‐SP (Figure S8, Supporting Information) was recovered, confirming the reversible nature of the EGO‐MC to EGO‐SP back‐isomerization in thin films.

**Figure 2:**
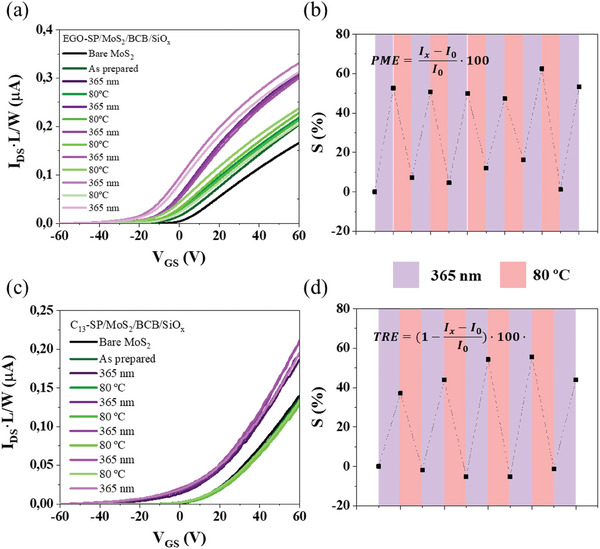
Transfer characteristics of MoS_2_/BCB/SiO_x_ after and before the deposition of the (a) EGO‐SP and (c) C_13_‐SP by spin‐coating on the MoS_2_ channel. Reversible modulation of drain‐source current of (b) EGO‐SP/MoS_2_/BCB/SiO_x_ and (d) C_13_‐SP/MoS_2_/BCB/SiO_x_ based FETs when subjected to cycles of UV‐irradiation (365 nm, violet shaded areas) and thermal annealing (80 °C, red shaded areas). The V_DS_ was 0.1 V, and the measurements were recorded inside the glove box to avoid the influence of the environmental humidity.

The SP molecule/MoS_2_ hybrid structures were investigated as an active layer in FETs, with the aim of exploring the influence of the SP derivative on the light‐responsiveness of both the electronic properties and the device's humidity‐response, especially in relation to SP/MC isomerization. The MoS_2_ FETs were fabricated by photolithography and thermal evaporation of Cr/Au (3/60 nm) on top of the bare SiO_x_ or BCB‐coated SiO_x_. Finally, the SP molecules were spin‐coated and thermally annealed at 80 °C for 10 min to promote the complete solvent evaporation and formation of the SP isomer. The transfer characteristics for all the devices are shown in **Figures** **2**a–d and S9 (Supporting Information), and the parameters extracted are reported in **Table** **1**.

**Table 1:** Summary of key parameters of FETs based MoS_2_ and heterojunction EGO‐SP/MoS_2_ and C_13_‐SP/MoS_2_.

	V_TH_ (V)	µ (cm^2^ V^−1^ s^−1^)	PME [%]^a)^	TRE [%]^b)^
MoS_2_/SiO_x_	−9.4	18.2	–	–
EGO‐SP/MoS_2_/SiO_x_	−14.1	19.8	27.0±2.5	–
EGO‐MC/MoS_2_/SiO_x_	−22.6	23.6	–	98.7±1.6
				
MoS_2_/BCB/SiO_x_	−6.8	16.7	–	–
EGO‐SP/MoS_2_/BCB/SiO_x_	−11.4	17.8	52.7±5.2	–
EGO‐MC/MoS_2_/BCB/SiO_x_	−20.8	27.0	–	91.7±6.0
				
MoS_2_/SiO_x_	6.5	16.3	–	–
C_13_‐SP/MoS_2_/SiO_x_	4.4	18.2	29.2±3.2	–
C_13_‐MC/MoS_2_/SiO_x_	−5.0	21.0	–	94.6±1.6
				
MoS_2_/BCB/SiO_x_	−3.8	14.0	–	–
C_13_‐SP/MoS_2_/BCB/SiO_x_	−5.7	14.2	46.9±7.8	–
C_13_‐MC/MoS_2_/BCB/SiO_x_	−15.1	22.9	–	96.6±2.1

^a)^The photo‐modulation efficiency (PME) values were calculated by estimating the percentage of UV‐increased I_DS_ versus initial I_DS_;

^b)^The thermo‐recovered efficiency (TRE) values were quantified by estimating the percentage of thermal recovery I_DS_ versus before UV exposure.

The devices based on bare MoS_2_ (black line) supported on SiO_x_ (Figure S9, Supporting Information) and BCB/SiO_x_ (Figure 2a,b) exhibited n‐type behavior with threshold voltage values (V_TH_) close to zero and electron mobilities (µ) up to 14 cm^2^ V^−1^ s^−1^. Upon deposition of the EGO‐SP (Figure 2a, **green line**) and C_13_‐SP (Figure 2b, **green line**) on the MoS_2_/BCB/SiO_x_, the V_TH_ displayed a small shift to negative values, indicating an electron doping effect due to the molecular adsorption.^[^ ^26^, ^41^, ^67^, ^68^ ^]^ Such shifts amount to −5 and −5 V for the EGO‐SP on MoS_2_/BCB/SiO_x_ and MoS_2_/SiO_x_, respectively, and −2 and −2 V for the C_13_‐SP on MoS_2_/BCB/SiO_x_ and MoS_2_/SiO_x_, respectively. It is noteworthy that the deposition of SP on MoS_2_/BCB/SiO_x_ and MoS_2_/SiO_x_ results in a slight increase in electron mobility of 1.1 and 1.6 cm^2^ V^−1^ s^−1^ for the EGO‐SP, respectively, and 0.2 and 1.9 cm^2^ V^−1^ s^−1^ for the C_13_‐SP, respectively.

After the irradiation with a 365 nm light during 10 min with an intensity of 0.7 mW·cm^−2^, the MoS_2_ transistor showed a significant improvement in the device performance. An increase of current in the transfer curves was observed over the entire range of the gate bias, and a further negative shift of the V_TH_. In particular, the EGO photo‐conversion of SP deposited on MoS_2_/BCB/SiO_x_ and MoS_2_/SiO_x_ yielding EGO‐MC was accompanied by a negative V_TH_ shift of −9 and −9 V, and an increase in the electron mobility of 9.2 and 3.8 cm^2^ V^−1^ s^−1^, respectively. Conversely, the isomerization into C_13_‐MC on MoS_2_/BCB/SiO_x_ and MoS_2_/SiO_x_ were characterized by a negative V_TH_ shift of −9 and −9 V, and mobility enhancement of 8.7 and 2.8 cm^2^ V^−1^ s^−1^, respectively. In terms of V_TH_ shift, both SiO_x_ and BCB/SiO_x_ supports shows same effect. Upon considering mobility, BCB maximizes the effect of SP molecules on MoS_2_.

Therefore, the electric fields emanated by the dipoles in the zwitterionic MC efficiently act as a local gate, by shifting the work function of MoS_2_, thus inducing a strong n‐type doping effect.^[^ ^41^ ^]^ As a result, the device field‐effect electron mobility and the V_TH_ were found to be strongly dependent on the state adopted by the SP molecule. The strong dipolar nature of zwitterionic MC molecules on the surface of chemical vapour deposition (CVD) MoS_2_ induces a reversible shift in the Fermi level (see Figure S10, Supporting Information), transitioning from 5.0 to 4.8 eV. This Fermi level shift was observable in devices as a notable n‐type doping effect. The analysis of the data in Figure 2c,d, and Figure S9 (Supporting Information) made it possible to quantify the photo‐modulation efficiencies (PMEs) and high thermo‐recovered efficiencies (TREs) of the EGO‐SP/MoS_2_ and C_13_‐SP/MoS_2_ hybrid structures. EGO‐SP/MoS_2_‐based devices exhibited PME values of 27±3% and 53±5% on SiO_x_ and BCB/SiO_x_ substrates, respectively. Differently, C_13_‐SP/MoS_2_‐based devices showed PME values of 29±3% and 47±8%. The higher PME observed when using BCB/SiO_x_ substrates can be attributed to the reduced density of traps at the MoS_2_/dielectric interface.^[^ ^38^ ^]^ Furthermore, the thermal reversibility of SP molecules/MoS_2_ heterostructure FET devices was estimated by monitoring the device response upon thermal annealing at 80 °C for 10 min. EGO‐MC/MoS_2_‐based devices exhibited TRE values of 99±2% and 92±6% on SiO_x_ and BCB/SiO_x_ substrates, respectively, while C_13_‐SP/MoS_2_‐based devices showed TRE values of 95±2% and 97±2% on SiO_x_ and BCB/SiO_x_, respectively. The excellent TRE indicates that it is possible to perform cycles of photo‐conversion without observing a significant photo‐degradation of the switching capability. Moreover, the consistent and high PME offers insights into the reversibility of the hybrid structure. Although the PME and TRE associated with photo‐switching of SP fluctuate, there was no evident degradation trend observed. The high stability in photo‐switching has previously been observed in graphene and MoS_2_ devices decorated with alkyl SP derivatives.^[^ ^41^ ^]^ It should be noted that the photo‐switching measurements were performed in a nitrogen filled glove‐box to avoid any influence of H_2_O and O_2_ in the environment which was previously observed when combining EGO‐SP with polymeric semiconductors.^[^ ^65^ ^]^

Notably, both EGO‐SP/MoS_2_ and C_13_‐SP/MoS_2_ systems displayed strikingly similar photo‐responsive properties, with robust thermal recovery which is independent on the substrate or side chain. After five cycles of photo‐switching, neither EGO‐SP/MoS_2_ nor C_13_‐SP/MoS_2_ exhibited considerable photo‐fatigue. The blank experiments carried out on bare MoS_2_ devices revealed the absence of photo‐response upon exposure to UV light or thermal treatment under inert atmosphere (Figure S11, Supporting Information). The increase of the current during the isomerization from SP to MC can be ascribed to enhancement in the molecular dipole moment of the isomer ruling the doping, which is in line with our previous report.^[^ ^41^ ^]^

**Figure 3:**
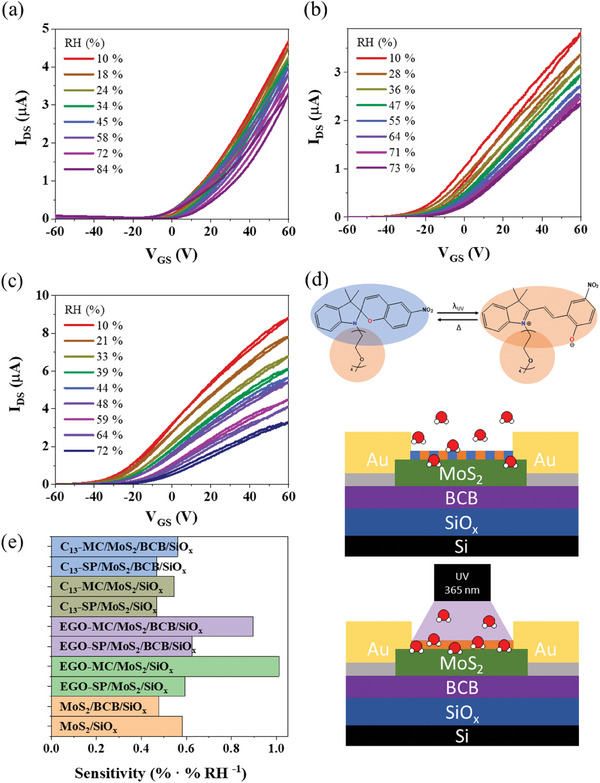
Transfer characteristics of the FET incorporating (a) MoS_2_/BCB/SiO_x_, (b) EGO‐SP/MoS_2_/BCB/SiO_x_, and (c) EGO‐MC/MoS_2_/BCB/SiO_x_ measured at different RH values. (d) Scheme of the water adsorption mechanism of the EGO‐SP(MC) at high humidity levels. (e) Humidity sensing response of all FETs based on MoS_2_ using the relative current change as gauge.

In order to assess the potential of our SP derivative/MoS_2_ hybrid structure based on FETs as humidity sensor, the performances of devices containing bare MoS_2_, EGO‐SP/MoS_2_ (**Figure** **3**; Figure S12, Supporting Information) and C_13_‐SP/MoS_2_ (Figure S13, Supporting Information) were analyzed at various relative humidity (RH) levels, by making use of a home‐made humidity chamber. At each change in RH, the samples were equilibrated for 5 min. Previous studies on the effect of the increasing RH on the performance of MoS_2_ transistors revealed a simultaneous decay of the current, increase of the hysteresis, and positive shift of the threshold voltage.^[^ ^61^, ^62^, ^69^ ^]^ The humidity range was selected to minimize the influence of leakage current. SiO_x_ is a hygroscopic material that exhibits a high humidity dependence as evidenced by an increase in leakage current with high moisture content reaching values up to 10–100 nA above 75–80% RH.^[^ ^70^ ^]^ Therefore, measurements were carried out at RH up to 75%. The minimum RH reached was 10%, primarily attributable to difficulties to stabilizing RH levels below this threshold, and the time required for stabilization the RH.

Upon increasing the RH from 10 to 70–75%, our devices based on bare MoS_2_/SiO_x_ (Figure S12a, Supporting Information) displayed a decay of ca. 37±4% of the initial current (at low humidity values), and a major positive shift of the V_TH_ of 16±2 V. The comparison of the forward and reverse curves reveals the presence of hysteresis, and the trends observed on the V_TH_ shifts are not easy to rationalize. Conversely, when a BCB layer was placed at the MoS_2_/SiO_x_ interface (Figure 3a) significantly lower drops of the initial current were observed 30±9%, in the same humidity range, along with a lower V_TH_ shift of 10±3 V. The coating of the SiO_x_ with BCB reduces the interference of the SiO_x_ on the humidity sensing. As a result, the MoS_2_/BCB/SiO_x_ devices exhibited negligible hysteresis, which simplifies the analysis of the V_TH_.

The electrical responses of the FETs to changes in the RH in the range from 10 to 70–75% RH after the deposition of the EGO‐SP and C_13_‐SP onto MoS_2_/SiO_x_ and MoS_2_/BCB/SiO_x_ were comparable to those observed for the bare MoS_2_ based devices. The decay of the current on the EGO‐SP/MoS_2_/SiO_x_ and EGO‐SP/MoS_2_/BCB/SiO_x_ were 35±3% and 38±2%, respectively. Conversely, for the C_13_‐SP we observed a slightly lower decay of the current which amounted to 30±2% and 32±2% for C_13_‐SP/MoS_2_/SiO_x_ and C_13_‐SP/MoS_2_/BCB/SiO_x_, respectively. In other words, the decoration of the MoS_2_ surface with EGO‐SP and C_13_‐SP did not significantly modify its humidity sensitivity (Figure 3b; Figures S12b and S13a,b, Supporting Information), with the subtle changes observed in the humidity response being in accordance with the small deviations in the measured CA on CVD MoS_2_ (Figure S14, Supporting Information) after the EGO‐SP deposition. In particular, CA measurement of CVD MoS_2_ indicated a hydrophobic nature of the surface (80.8°), in agreement with values reported in the literature.^[^ ^71^, ^72^ ^]^ Its functionalization with EGO‐SP leads to a CA = 82.7°.

After the UV irradiation, the transistor exhibited a strong n‐type doping. Figure S12c (Supporting Information) and Figure 3c show that the decay of the current in FET comprising EGO‐MC/MoS_2_/SiO_x_ and EGO‐MC/MoS_2_/BCB/SiO_x_ when increasing the RH from 10 to 70–75% amounts to as much as 61±4% and 59±3%, respectively. Such a high magnitude can be ascribed to the strong affinity of the hydrophilic EGO‐MC for water molecules. The CA of the EGO‐MC/CVD MoS_2_ corresponds to 71.5°after the UV exposure and it was found to quickly decrease within the timescale of a few tens of seconds (Figure S14, Supporting Information), reaching 66.5°after 2 min. The swift decay in CAs can be attributed to the water adsorption phenomenon by the EGO‐MC layer, contrasting the behavior typically observed in bare MoS_2_.^[^ ^73^ ^]^

Conversely, Figure S13c,d (Supporting Information) reveals that the photo‐isomerization of C_13_‐SP to form C_13_‐MC is characterized by a lower decay of current of 38±5% and 34±2% when supported on MoS_2_/SiO_x_ and MoS_2_/BCB/SiO_x,_ respectively. Thus, the conversion of the EGO‐SP to EGO‐MC is accompanied by an enhancement in the sensitivity to RH changes, as a result of the zwitterionic nature of the molecule. Such a sensitivity is overall greater compared to C_13_‐SP and C_13_‐MC because of the enhanced hygroscopic character of the ethylene glycol versus alkyl side chain (Figure 3d).

Figure 3e reports the sensitivity (*S*) of the different humidity sensors based on MoS_2_ and SP/MoS_2_ transistors which has been quantified by estimating the slope of the current decrease in the RH range spanning from 10 to 75% (Figure S15, Supporting Information). It is worth noting that bare MoS_2_ on SiO_x_ and BCB/SiO_x_ substrates exhibit different *S*. The hygroscopic nature of the SiO_x_ affects the sensitivity to humidity changes: the use of SiO_x_ supports displayed the highest sensitivity (0.6% · (%) RH^−1^) compared to BCB/SiO_x_ (0.5% · (%) RH^−1^). Nevertheless, the hydrophobic character of BCB was instrumental to provide a high‐quality hydroxyl‐free interface, limiting the water adsorption at the dielectric/MoS_2_ interface and onto the channel MoS_2_ thereby improving the device stability in air, as evidenced by the lowest hysteresis, and the easier processing of the data to develop the humidity sensor (Figure 3a–c). Conversely, the MoS_2_/SiO_x_ exhibited a major hysteresis (Figure S12, Supporting Information) due to the large density of interfacial traps.

Following, the RH sensitivity (Figure 3e) of the MoS_2_ was modulated via the physisorption of molecules possessing tuneable hydrophilic/hydrophobic properties. In particular, the gain in the sensitivity can be quantified by estimating the ratio between the sensitivities in two specific cases. For example, the increase in sensitivity upon isomerization of the SP to MC in EGO‐SP corresponds to S_MC_/S_SP_, which amounts to 1.0/0.6 = 1.7 for the devices based on EGO‐SP/MoS_2_ on SiO_x_ whereas a value of 0.9/0.6 = 1.5 was determined when using BCB/SiO_x_ as support. Conversely, S_MC_/S_SP_ on the C_13_‐SP/MoS_2_ resulted in 0.55/0.47 = 1.2 and 0.56/0.47 = 1.2, for devices based on EGO‐MC/MoS_2_ on SiO_x_ and BCB/SiO_x_, respectively. The big gain observed for the EGO‐SP/EGO‐MC is a result of joint effect of the ethylene glycol hydrophilic chains and strong change in dipole of the headgroup, both strongly interacting with water molecules. Conversely, the hydrophobic alkyl chains and the strong dipole of the C_13_‐MC did not provide a similar strong enhancement in water adsorption. The Fourier transform infrared (FT‐IR) measurements carried out under different humidity conditions provide strong evidence of the ability of EGO‐SP and C_13_‐SP to interact with water molecules. In particular, both EGO‐SP and C_13_‐SP showed a small absorption band ≈3500 cm^−^¹ due to water adsorption and hydrogen bonding (Figure S16). For the former molecule, after photo‐isomerization to EGO‐MC the intensity of this band increased significantly. The joint effect of the hydrophilic ethylene glycol and the major dipole change in the photoresponsive core of the molecule strongly modify the local wettability of the surface upon isomerization from EGO‐SP to EGO‐MC, enhancing water adsorption. These phenomena were not observed in the case of C_13_‐SP (and C_13_‐MC). In such a case the band ≈3500 cm⁻¹ was found to be little sensitive to the occurrence of the isomerization from SP to MC even at high humidity levels indicating that the observed increase in humidity sensitivity is solely due to the formation of a strong dipole upon isomerization of the molecular core into the zwitterionic merocyanine form. Finally, it is worth stressing that the gain on the sensitivity (S_MC_/S_MoS2_) of the EGO‐MC produced by the decoration of the MoS_2_ were 1.0/0.6 = 1.7 and 0.9/0.5 = 1.8 on the SiO_x_ and BCB/SiO_x,_ respectively.

Typically, humidity sensors based on MoS_2_ transistor exhibit poor performance or unclear trends when exposed to humid air. By making use of simple passivation of the SiO_x_, our approach of SP/MoS_2_ on BCB/SiO_x_ overcomes key intrinsic bottlenecks of MoS_2_ humidity sensors such as the uncontrolled hysteresis^[^ ^74^, ^75^ ^]^ and the narrow operational range of the humidity sensors based on MoS_2_ transistor (10 to 40% RH).^[^ ^61^ ^]^ By and large, the passivation of the SiO_x_ is an attractive approach for the development of multifunctional sensors because it decouples the impact of silanols on the device response.

The light‐induced photo‐switching triggers a wettability change of the SP/MoS_2_ interface, in turn, worsens the optoelectronic properties of the MoS_2_ semiconductor due to water's known role as an electron trap in MoS_2_. There are examples in the literature where invasive external stimuli, such as strain, have been shown to affect the sensitivity to humidity changes in FETs based on MoS_2_ monolayer.^[^ ^76^ ^]^ However, our approach has the advantage of relying on non‐invasive inputs enhancing the humidity sensitivity of MoS_2_ transistors.

The reversible nature of water adsorption onto the MoS_2_ surface was assessed upon successive device exposure to humid and dry air. It was found that the initial performance of the MoS_2_ devices could be partially restored after 1 h of flushing a gentle stream of N_2_ to remove the water (Figure S17, Supporting Information), recovering 96.3% and 99.8% of the initial current, for the MoS_2_/SiO_x_ and MoS_2_/BCB/SiO_x_, respectively.

Finally, in order to evaluate the effect of the thermal back‐isomerization from MC to SP occurring at room temperature on the sensitivity in humidity sensing, we investigated the characteristics of our devices over the time scale of a few hours when exposed to low humidity (<5% RH) (Figure S18, Supporting Information). The bare MoS_2_ and EGO‐SP/MoS_2_ exhibited stable device outputs over time. In contrast, EGO‐MC/MoS_2_ transistors displayed a slight drop in current (<5%) after 2 h of exposure to dry air, as a result of the back‐isomerization from the MC to SP isomer occurring at room temperature. Such result is a strong confirmation that the response to humidity changes we have reported above is due to the adsorption of water molecules onto the channel material, whereas the impact of the exposure to oxygen on the device stability was negligible over the short time frame of a few hours.

## 3 Conclusion

In summary, we developed an optically switchable MoS_2_ humidity sensor by dressing 2DS flakes with a light‐responsive spiropyran ultrathin layer. Upon optimizing the hydrophobic nature of the support and the hydrophilic character of the spiropyran derivative, high sensitivity in the sensing events could be attained, reaching values of 0.5% · (%) RH^−1^ for the bare MoS_2_ and 0.5% · (%) RH^−1^ for the bare EGO‐SP/MoS_2_. Such a sensitivity can be further boosted to 0.9% · (%) RH^−1^ upon triggering the photo‐isomerization from SP to MC, thereby enhancing further the hydrophilicity of the surface.

The demonstration of multi‐responsive sensing capability of organic/2D semiconductor hybrids structure is a milestone toward the development of an unprecedented class of reconfigurable chemical sensors combining high sensitivity and selectivity with tuneable performance.

## 4 Experimental Section

### Materials

The MoS_2_ bulk crystal semiconductors were purchased from SPI. The CVD few layers MoS_2_ continuous films on SiO_x_ were purchased from 6 Carbon. The BCB polymer, divinyltetramethyl‐isiloxanebis(benzocyclobutene), was purchased from Dow Chemical (CYCLOTENE) and diluted with mesitylene solution (1:4 volume ratio). The anhydrous toluene, methylene, ethanol, isopropanol and acetone were purchased from Sigma–Aldrich. EGO‐SP and C_13_‐SP were synthesized according to previous studies, with some modifications.^[^ ^65^ ^]^ The 270‐nm thermally oxidized silicon wafers were purchased from Fraunhofer Institute IPMS (n‐doped Si).

### Device Fabrication

MoS_2_ flakes were mechanically exfoliated from a bulk crystal using blue Scotch tape (purchased from Nitto), and then a polydimethylsiloxane (PDMS) film (purchased from Gel‐Pak) was used to transfer the MoS_2_ flakes from the Scotch tape on SiO_x_ substrates or BCB/SiO_x_ substrates. The BCB/SiO_x_ was prepared following the protocol reported by the Y. Zhao et al.^[^ ^38^ ^]^ 120 nm of BCB polymer was spin‐coated onto the SiO_x_ and cross‐linked by post‐annealing at 290 °C (10 min inside the glovebox).

The top‐contact source and drain electrodes were manufactured by photolithography and a Cr (3 nm) and Au (60 nm) layer were deposited by thermal evaporation. Then, the substrates were cleaned with acetone and isopropanol and then dried under nitrogen flow. Finally, the MoS_2_ transistors were annealed at 140 °C for 8 h in a high‐vacuum chamber to improve the contacts and remove adsorbents, such as water or organic solvents.

In order to fabricate the SP/MoS_2_ heterojunction, a thin layer of EGO‐SP (or C_13_‐SP) was spin‐coated (1200 rpm for 60 s) onto the MoS_2_ FET. Subsequently, a thermal treatment to remove the solvent residue was carried out at 80 °C for 10 min. All the devices were kept in a nitrogen‐filled glovebox during the processing and annealing of the SP/MoS_2_ heterojunction.

### Characterization

UV–vis absorption spectra and isomerization kinetics of the SP molecule on quartz substrates were recorded at room temperature with a JASCO V650 spectrophotometer. Fourier transform infrared (FT‐IR) spectra of the SP molecule on quartz substrates were recorded using a FT‐IR 4700 Fourier Transform Infrared Spectrometer (JASCO) equipped with ATR Diamond. The CA measurements were conducted using a Krüss DSA 100 instrument. A 4 µL droplet of Milli‐Q water was deposited onto SiO_x_ and BCB/SiO_x_ substrates before and after printing the SP molecules, as well as after UV treatment of the SP molecules on SiO_x_ and BCB/SiO_x_ substrates.

The AFM measurements were carried out using a Bruker Dimension Icon microscope in ambient conditions, operating in tapping mode and using TESPA‐V2 tips with spring constant k = 42 N·m^−1^.

All light‐induced photo‐switching characterizations were conducted according to the following protocol: Quartz substrates were irradiated vertically with a UV light source (365 nm), emitting 0.7 mW·cm^−2^ for 10 min, using a Polychrome V monochromator (Till Photonics). To recover the SP form, the heating process was carried out using a hot plate set at 80 °C for 10 min.

### Electrical Characterization

The bottom‐gate top‐contact devices were electrically characterized by a Keithley dual‐channel 2636 A source meter in a three‐terminal configuration.

The linear carrier mobility µ was determined by the following equation:

(1) $$\mu \;=\;\frac{\partial I_{DS}}{\partial V_{GS}}\cdot \frac{L}{W\cdot C_{i}}\cdot \frac{1}{V_{DS}}$$

where L and W are the channel length and width and C_i_ is the dielectric capacitance of the material, the C values of 270 nm SiO_x_ and 120 nm/270 nm BCB/SiO_x_ were 12.79 and 6.32 nF·cm^−2^, respectively.

For light‐induced photo‐switching, a Polychrome V monochromator (Till Photonics) irradiated vertically the FETs with a UV light source (365 nm) emitting 0.7 mW·cm^−2^ for 10 min. The heating was performed using a hot plate set at 80 °C for 10 min. The experiments were carried out inside the glove box to mitigate the effects of atmospheric humidity.

The heterostructure device's response to changes in the RH was studied in a home‐made humidity chamber including commercial sensors (SHT31 Smart Gadget) for monitoring/controlling humidity and temperature, and equipped with a commercial humidifier. The heterostructure devices were fixed on PCB board, and connected with 80 µm thick gold wires.

## Conflict of Interest

The authors declare no conflict of interest.

## Author Contributions

A.T. and P.S. conceived the experiments and designed the study. A.T. performed all optoelectrical, AFM measurement, and UV–vis characterization. W.D performed the synthesis of the SP derivatives. Y.J. performed the Raman measurement and helped with the devices characterization. A.T and B.H. performed the CA measurement. B.H. helped with the fabrication of the devices. All authors discussed the results and contributed to the interpretation of data. A.T. and P.S. co‐wrote the paper with input from all co‐authors.

## Supporting information



Supporting Information

## Data Availability

The data that support the findings of this study are available from the corresponding author upon reasonable request.
