# Supplementary material for: Light‐Modulated Humidity Sensing in Spiropyran Functionalized MoS2 Transistors
Source: Small. 2024 Sep 12;22(25):2404633. doi: 10.1002/smll.202404633 (PMC13137231; doi:10.1002/smll.202404633)
Supplement: Supplementary file 1 — Supporting Information [file SMLL-22-2404633-s001.docx]

Supporting Information

Light-Modulated Humidity Sensing in Spiropyran functionalized MoS_2_ Transistors

Adrián Tamayo, Wojciech Danowski, Bin Han, Yeonsu Jeong, Paolo Samorì*


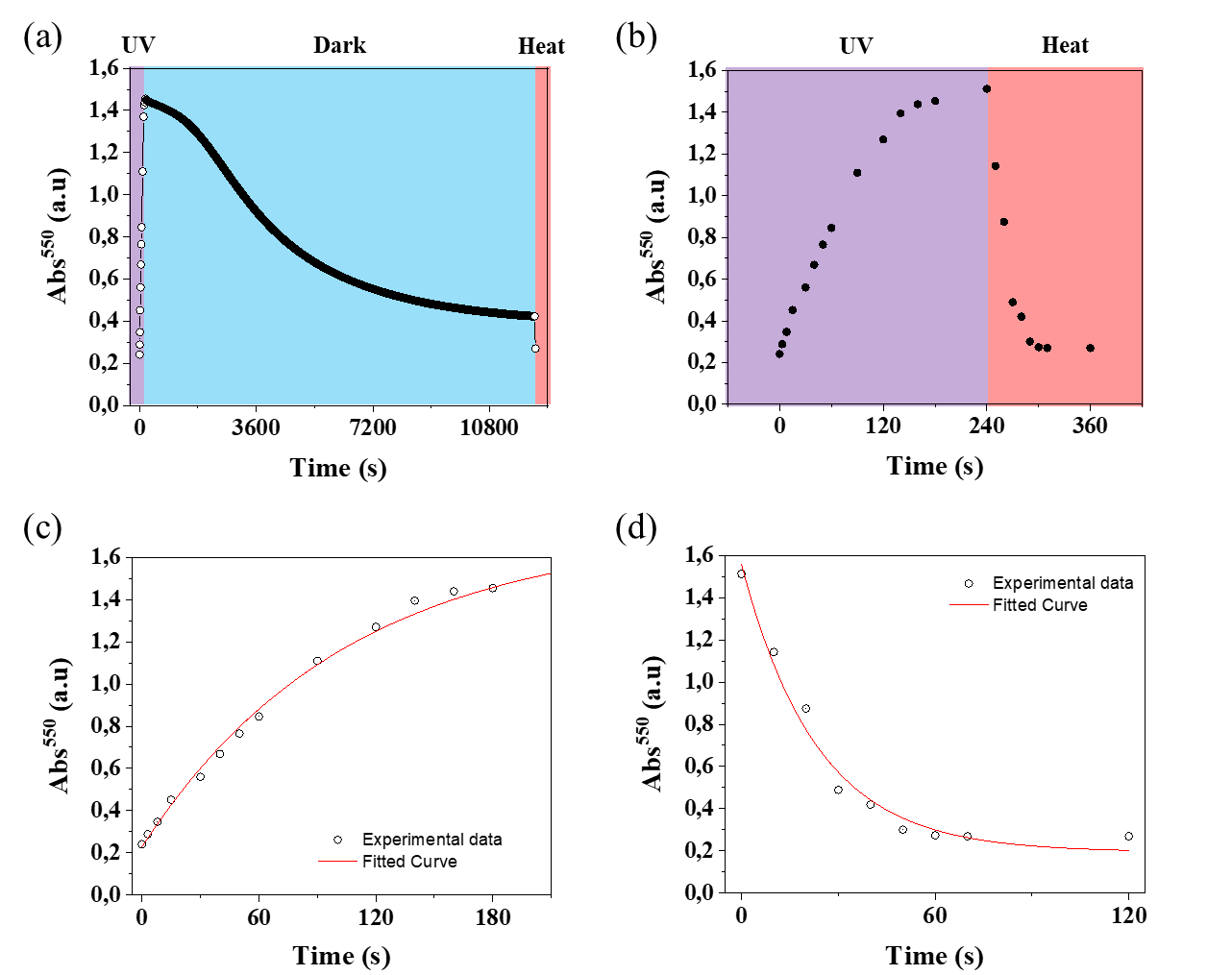


**Figure S1.** Kinetics of the photo-isomerization of EGO-SP to EGO-MC by plotting the absorbance changes over time at 550 nm, and its reversibility under different conditions: (a) isomerization with UV irradiation, followed by dark conditions at room temperature, and subsequently heating at 80 °C, (b) isomerization with UV irradiation and subsequent heating at 80 °C. Fitting of the (c) EGO-SP to EGO-MC isomerization and (d) EGO-MC to EGO-SP isomerization using a simple exponential equation.


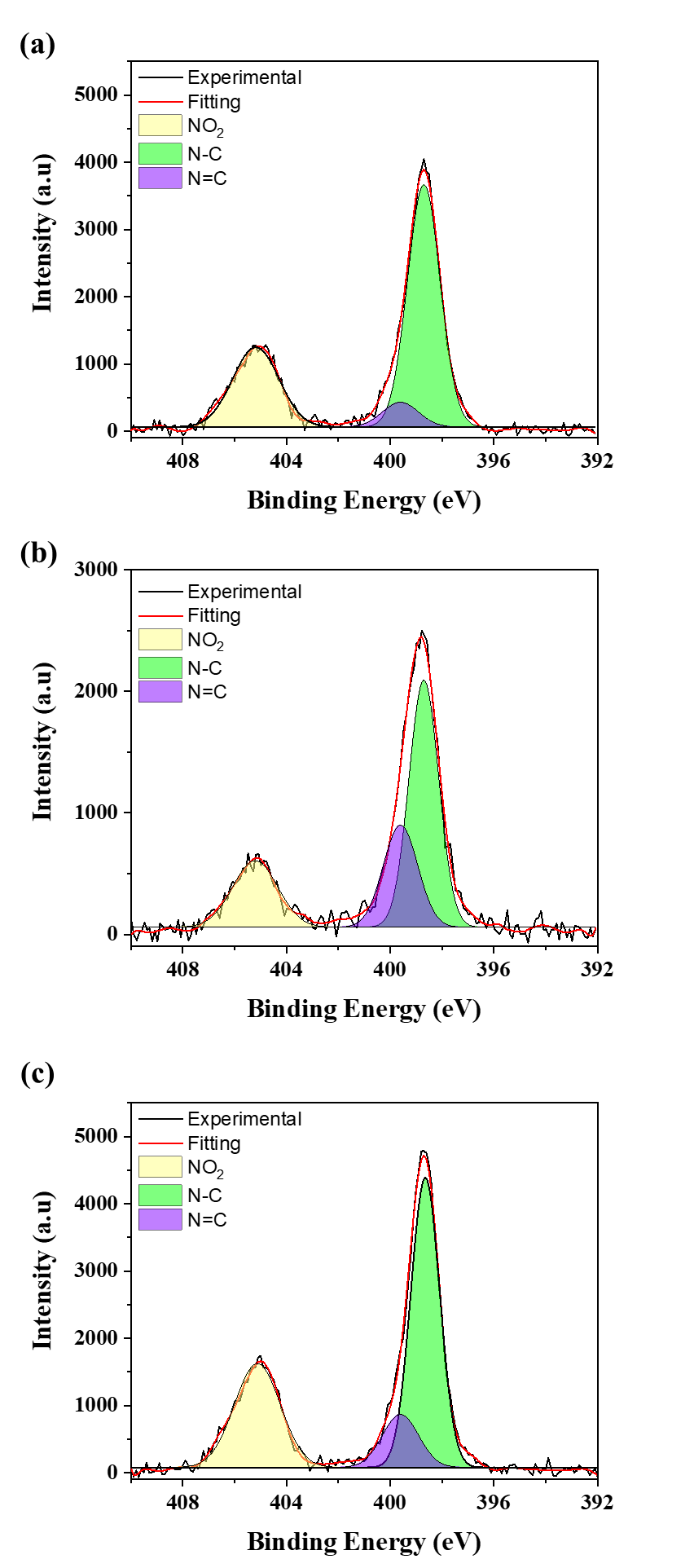


**Figure S2**. XPS measurements. N_1s_ core-level spectra measured on the same spin-coated thin film on SiO_x_ (a) as prepared, (b) after UV irradiation, and (c) after subsequent thermal annealing at 80 ºC. Each spectrum is characterized by multiple peaks fitted by different components, corresponding to the different N hybridization.


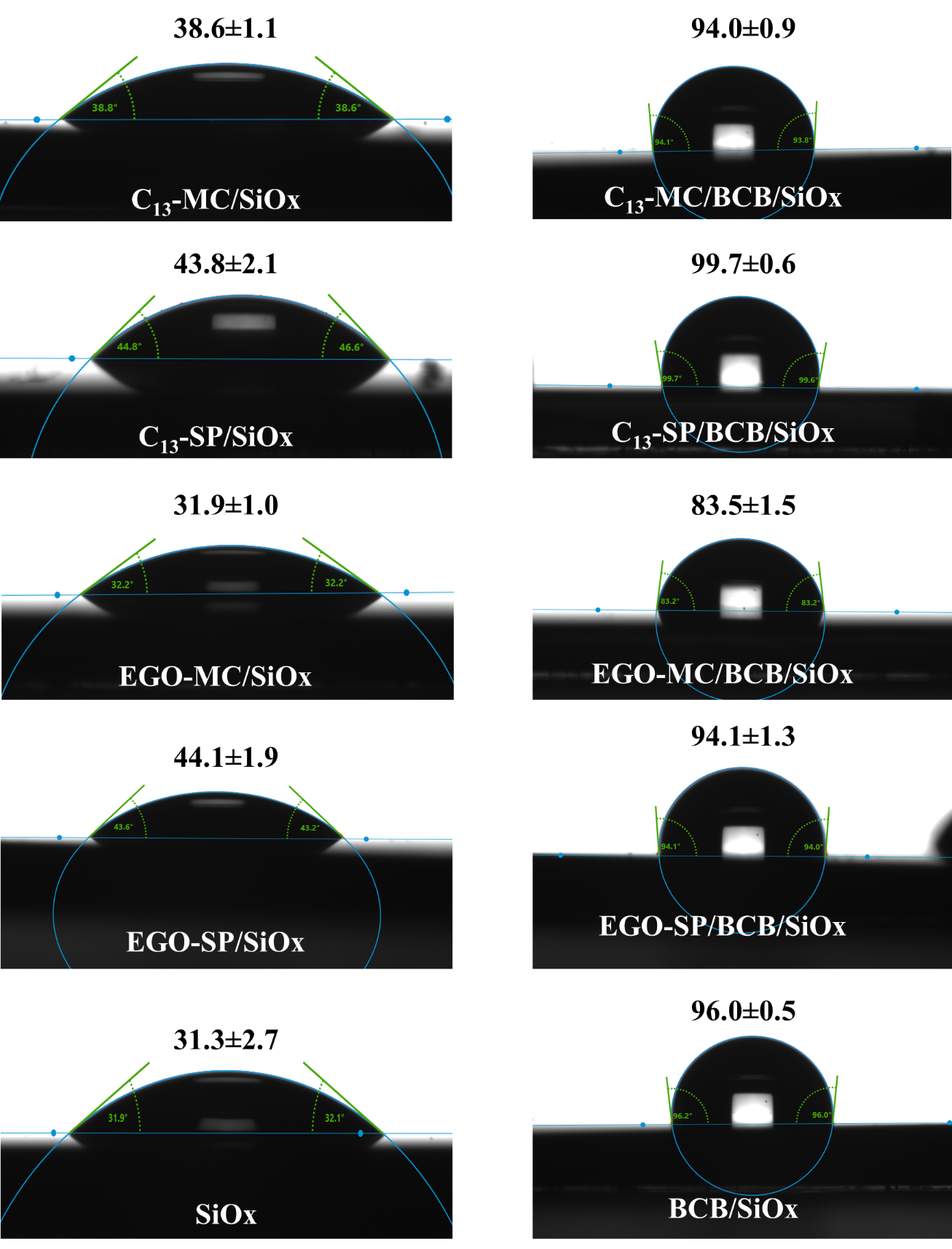


**Figure S3**. Contact angle measurements. Images of a water droplet on the surfaces of pristine SiO_x_ and BCB/SiO_x_, and SiO_x_ and BCB/SiO_x_ coated with EGO-SP and C_13_-SP after and before the UV irradiation.


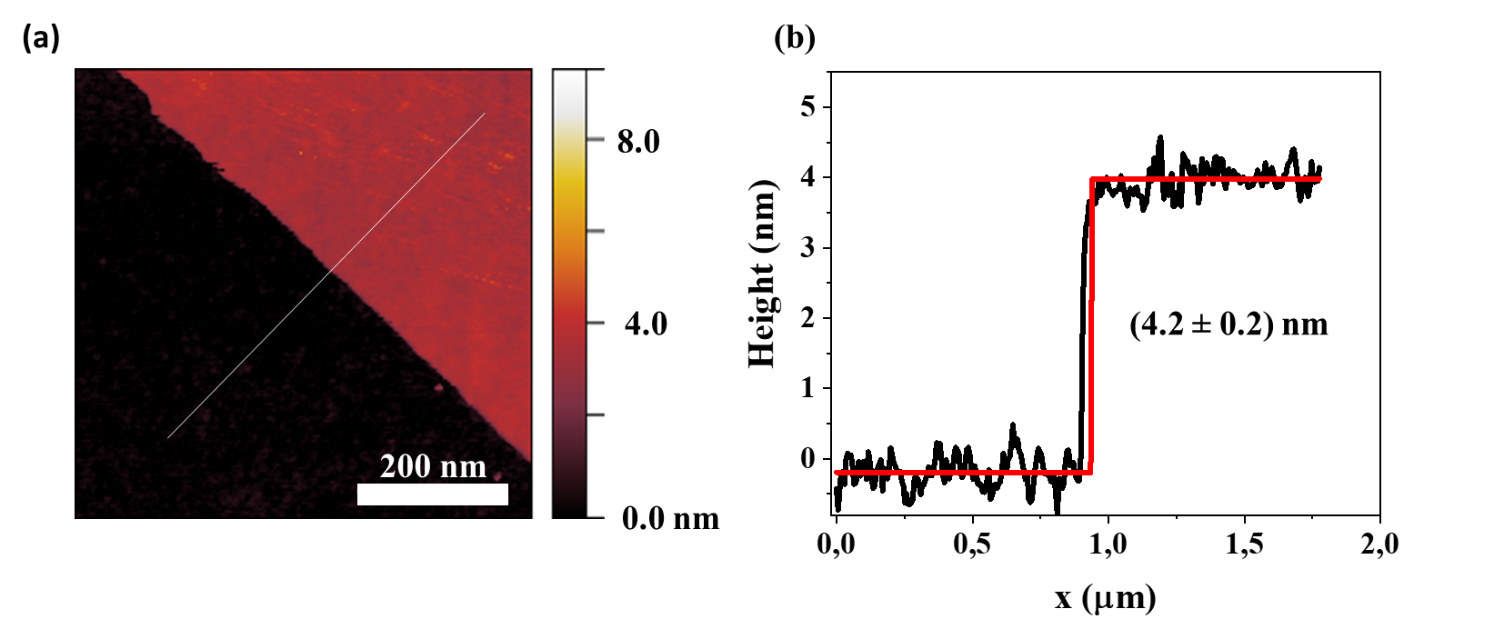


**Figure S4.** (a) AFM topographical images, and (b) topographical profile estimating the MoS_2_ flake thickness.


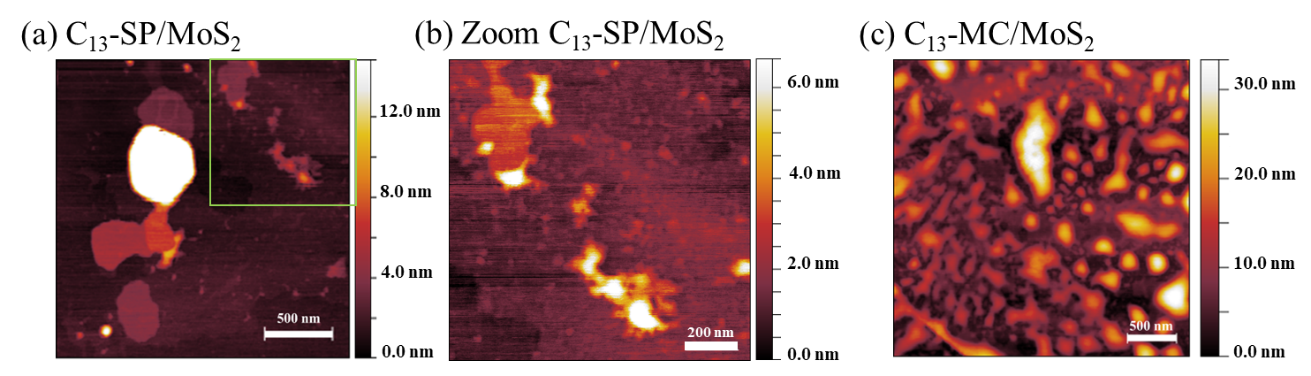


**Figure S5.** AFM topographical images of (a) C_13_-SP/MoS_2_ and its (b) zoom of the green area, as well as (c) C_13_-MC/MoS_2_.


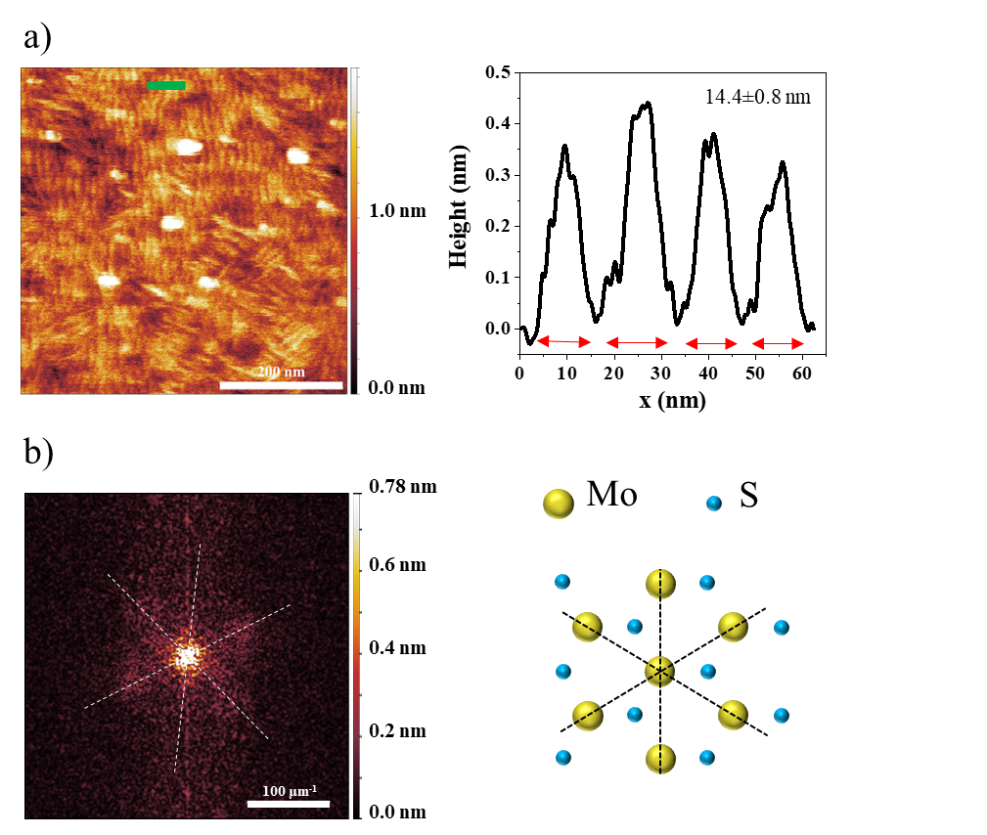


**Figure S6**. (a) AFM topographical image of the lamellar structure of EGO-SP on top of the MoS_2_ flakes with the topographical profile recorded along the green line of the lamellar EGO-SP structure. The 2D Fourier Transform (2D-FFT) shown in (b) reveals a hexagonal pattern following the geometry of the MoS_2_ lattice.


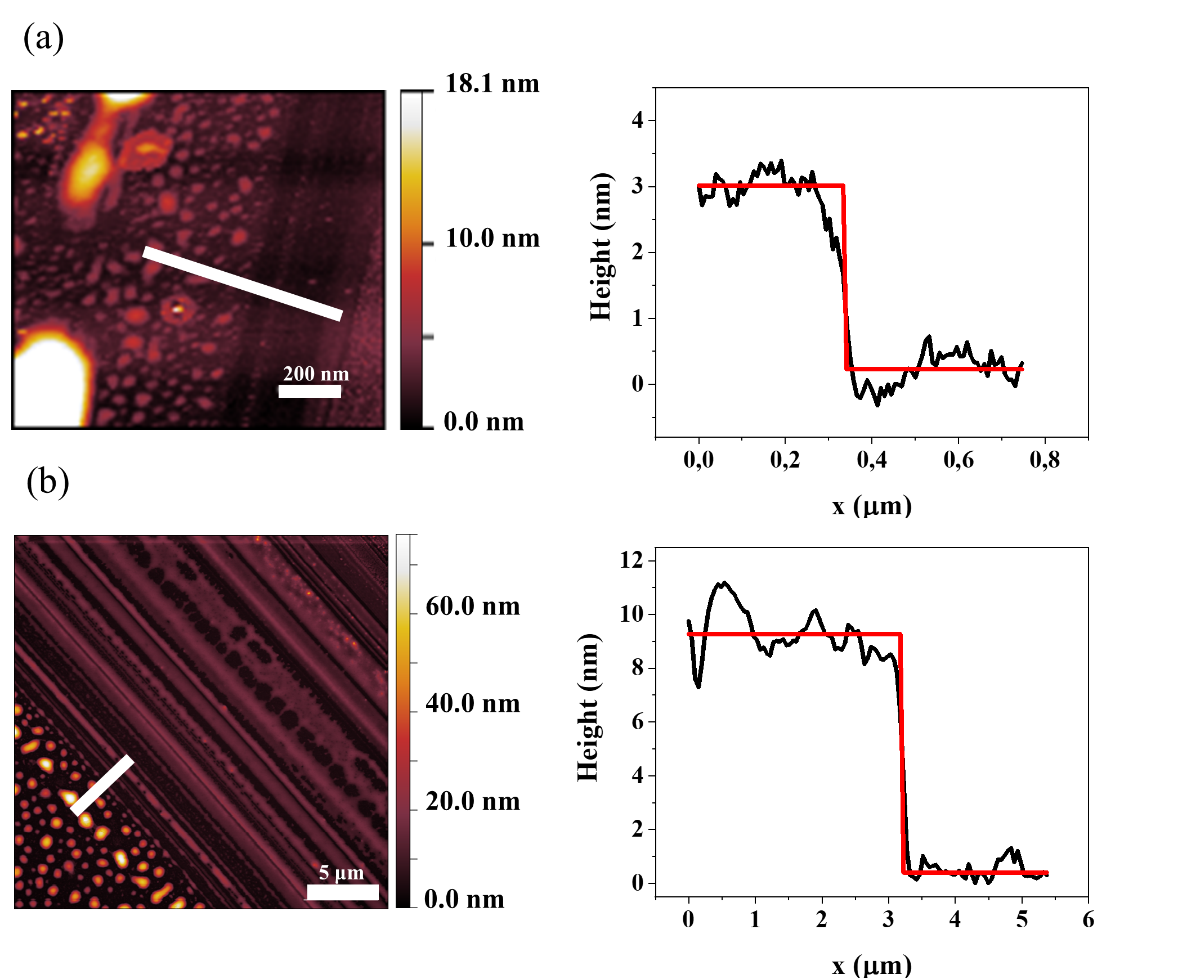


**Figure S7**. AFM topographical image and topographical profile of (a) EGO-SP and (b) C_13_-SP after molecular physisorption and thermal treatment at 80 ºC during 10 min. The white line marked in the images corresponds to the location where the profiles have been traced.


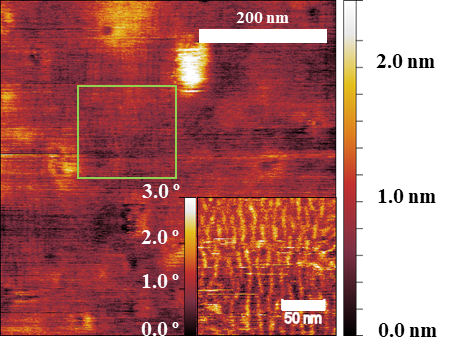


**Figure S8**. AFM topographical image of EGO-MC/MoS_2_ after a thermal treatment at 80 ºC during 10 min to get the EGO-MC form. The green square marked region corresponds with the zoom area in which the phase image is displayed.


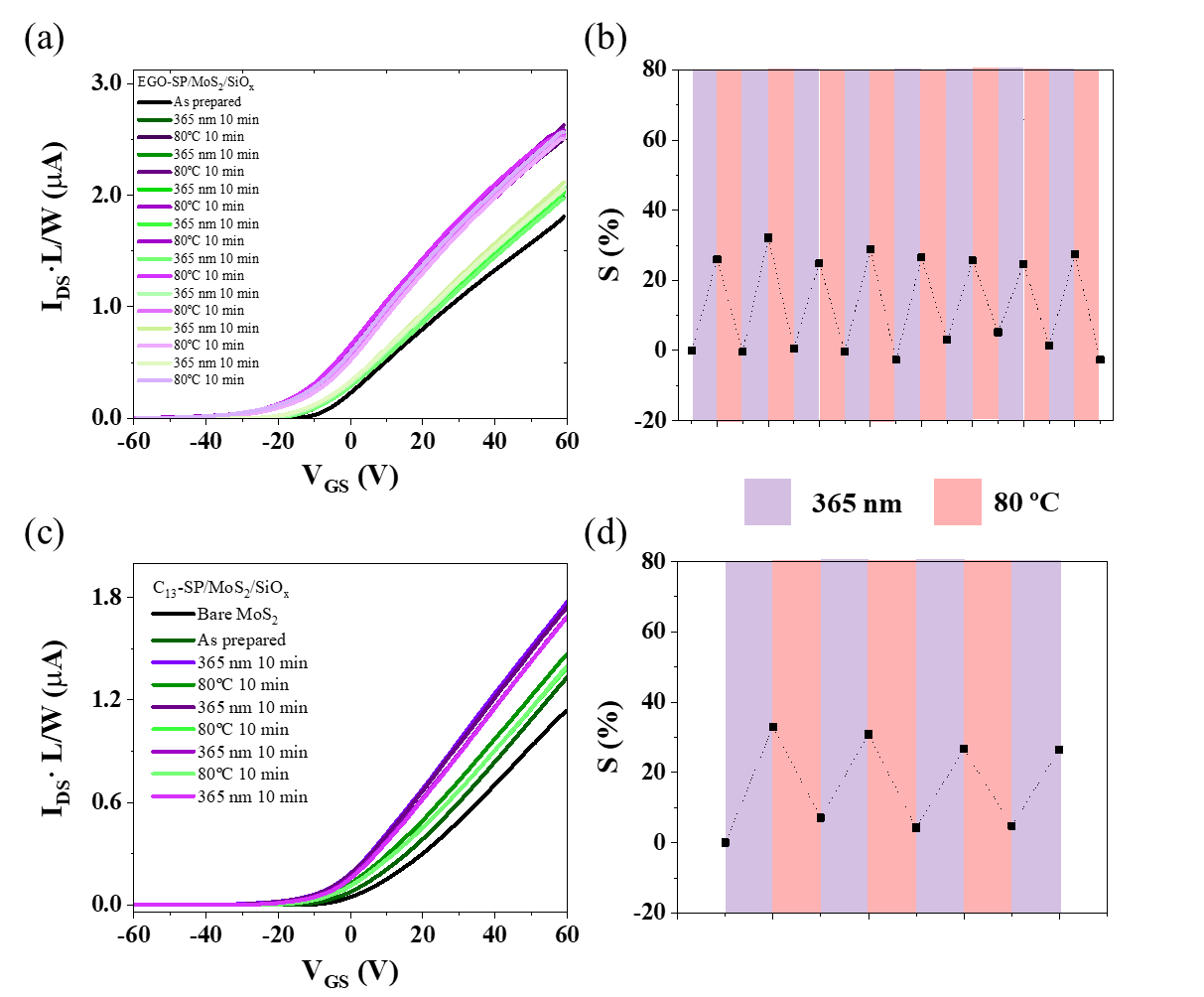


**Figure S9**. Transfer characteristics of MoS_2_/SiO_X_ after and before the deposition by spin coating of the (a) EGO-SP and (c) C_13_-SP on the MoS_2/_SiO_x_ transistor. Reversible modulation of drain-source current of (b) EGO-SP/MoS_2_/SiO_x_ and (d) C_13_-SP/MoS_2_/SiO_x_ based FETs over UV-irradiation cycles (365 nm, violet shaded areas) and thermal treatment (80 ºC, red shaded areas). The V_DS_ was 0.1 V, and the measurements were taken inside the glove box to avoid the impact of the humidity.


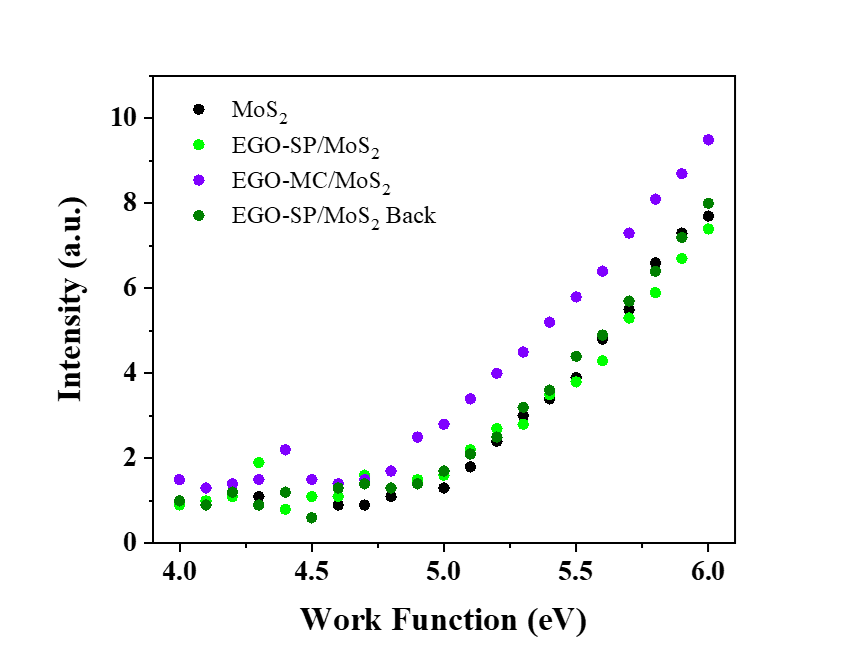


**Figure S10.** a) PYSA spectra of MoS_2_, EGO-SP/MoS_2,_ EGO-MC/MoS_2_ and EGO-SP/MoS_2._


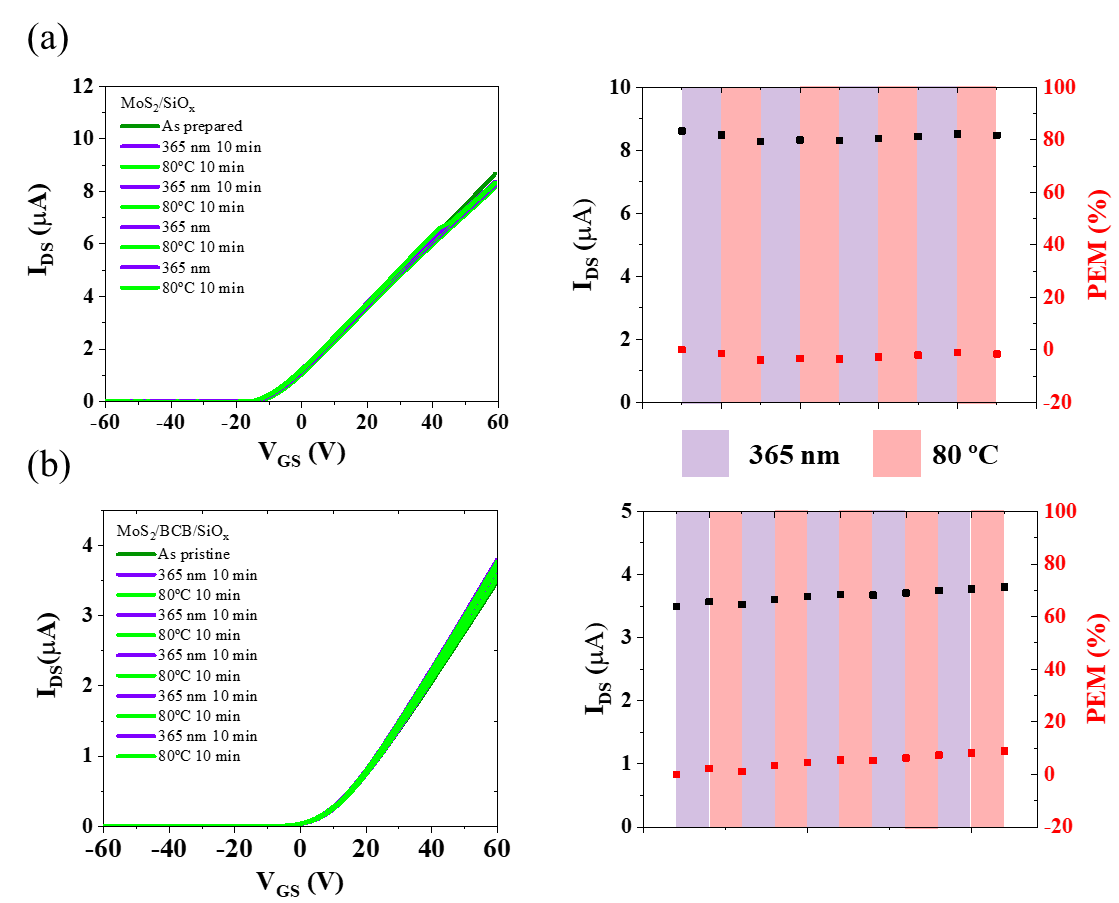


**Figure S11**. Transfer characteristics of (a) MoS_2_/SiO_x_ and (c) MoS_2_/BCB/SiO_x_ after exposure to 365 nm and 80 °C. (b) I_DS_ change and relative I_DS_ change of (b) MoS_2_/SiO_X_ and (d) MoS_2_/BCB/SiO_x_ after exposure to 365 nm, and 80 °C.


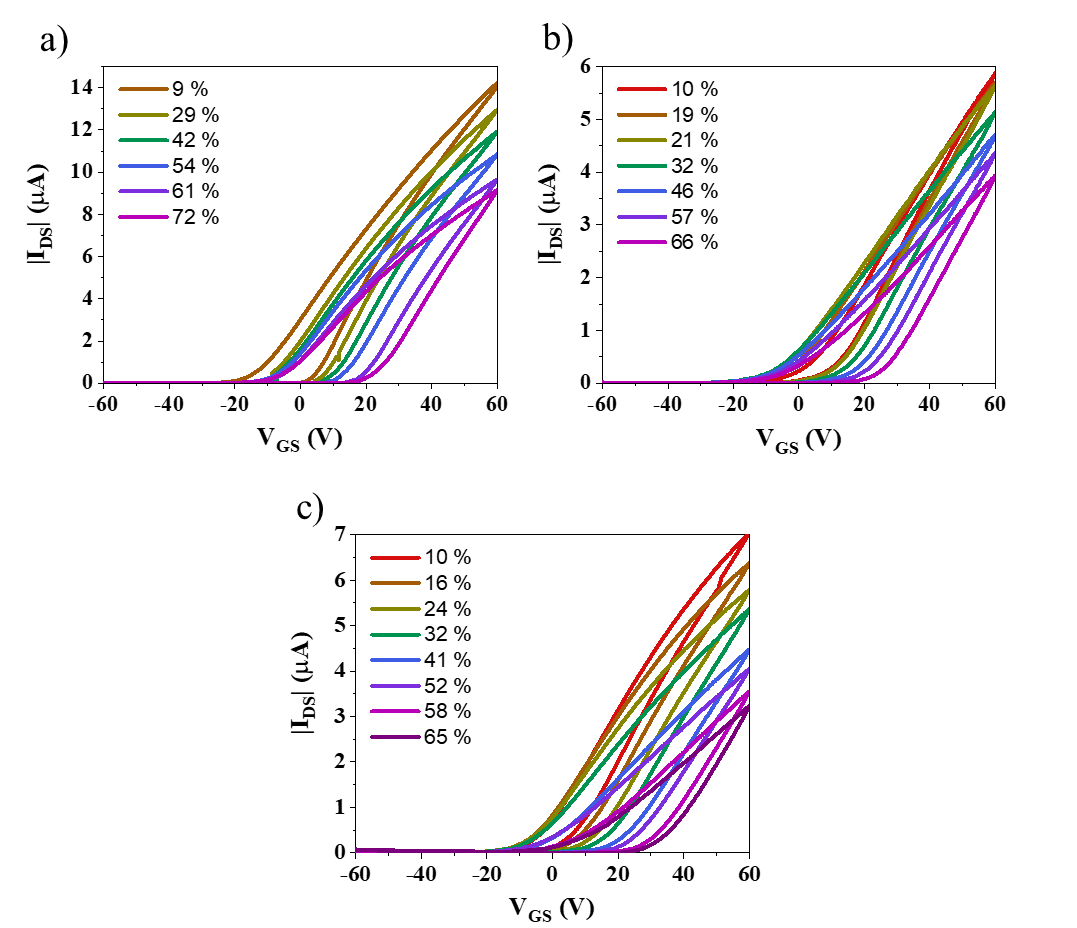


**Figure S12**. Transfer characteristics of the (a) MoS_2_/SiO_x_, (b) EGO-SP/MoS_2_/SiO_x_ and (c) EGO-MC/MoS_2_/SiO_x_ measured under different RH levels.


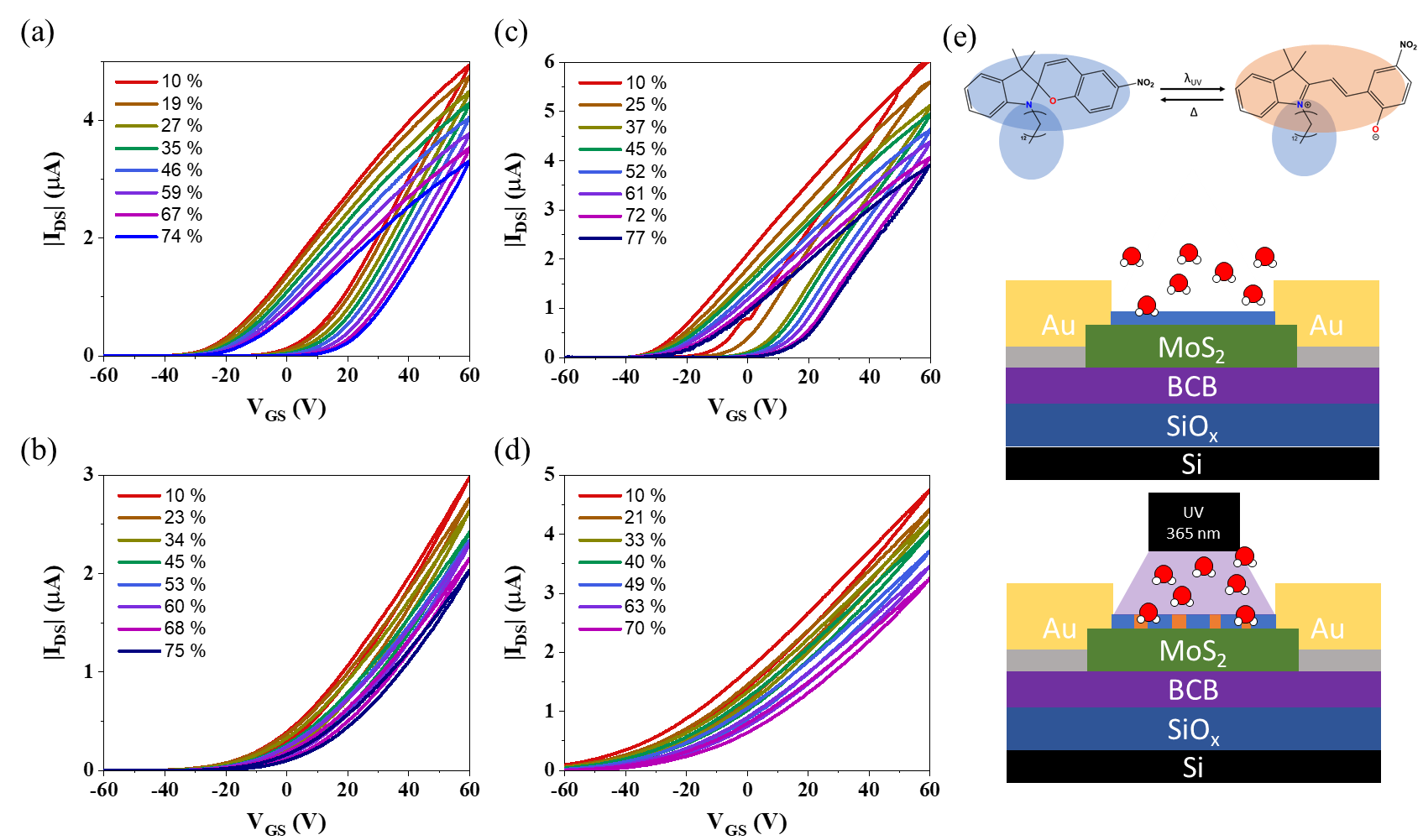


**Figure S13.** Transfer characteristics of the (a) C_13_-SP/MoS_2_/SiO_x_, (b) C_13_-SP/MoS_2_/BCB/SiO_x_, (c) C_13_-MC/MoS_2_/SiO_x_, and (d) C_13_-MC/MoS_2_/BCB/SiO_x_, measured under different RH levels. (e) Schema of the water absorption mechanism of the EGO-SP(MC)/MoS_2_/BCB/SiO_x_ at high humidity levels.


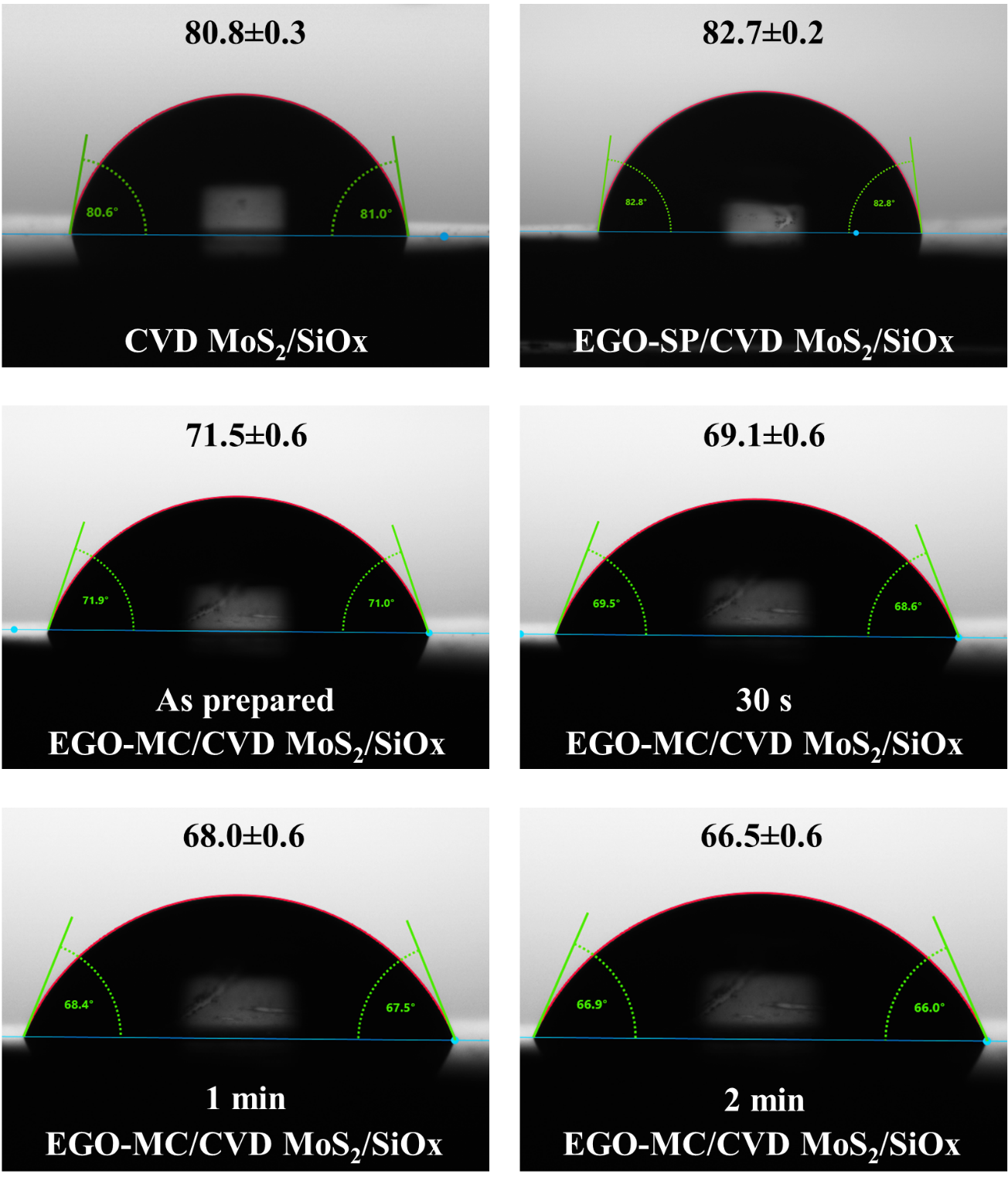


**Figure S14**. Contact angle measurements. Images of a water droplet on the surfaces of pristine MoS_2_/SiO_x_ and MoS_2_/SiO_x_ coated with EGO-SP after and before the UV irradiation. After the UV irradiation, a fast change of the measured contact angles was observed, on the timescale of a few minutes.

**
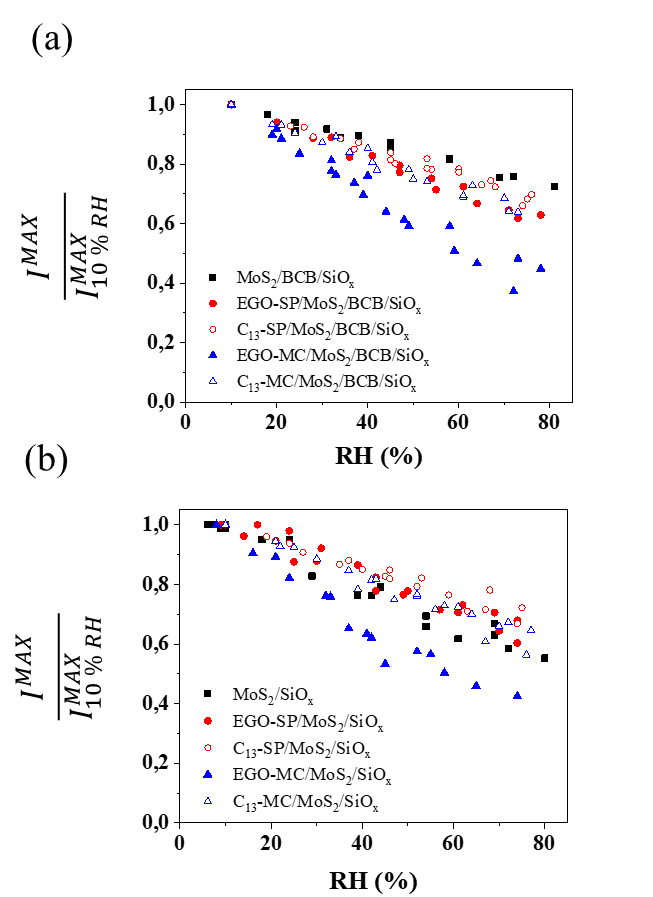
**

**Figure S15.** Relative source-drain current (at V_GS_ = 60 V) with respect to the current at % RH = 10, as a function of % RH for three different devices for films supported on (a) BCB/SiO_x_ and (b) SiO_x_.


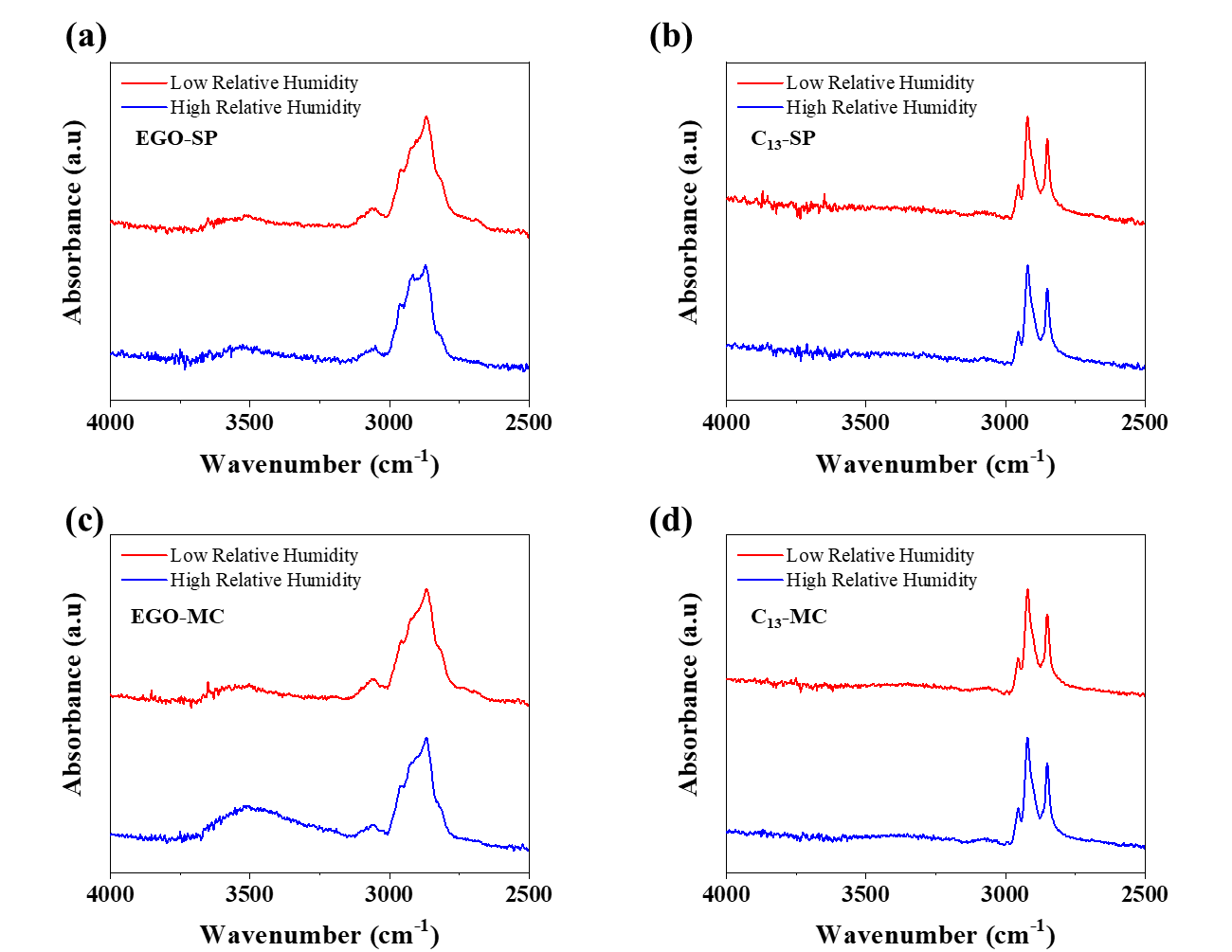


**Figure S16.** FT-IR spectra of (a) EGO-SP, (b) C13-SP, (c) EGO-MC, and (d) C13-MC layer physisorbed onto a quartz substrate before and after exposure to a high humidity environment.


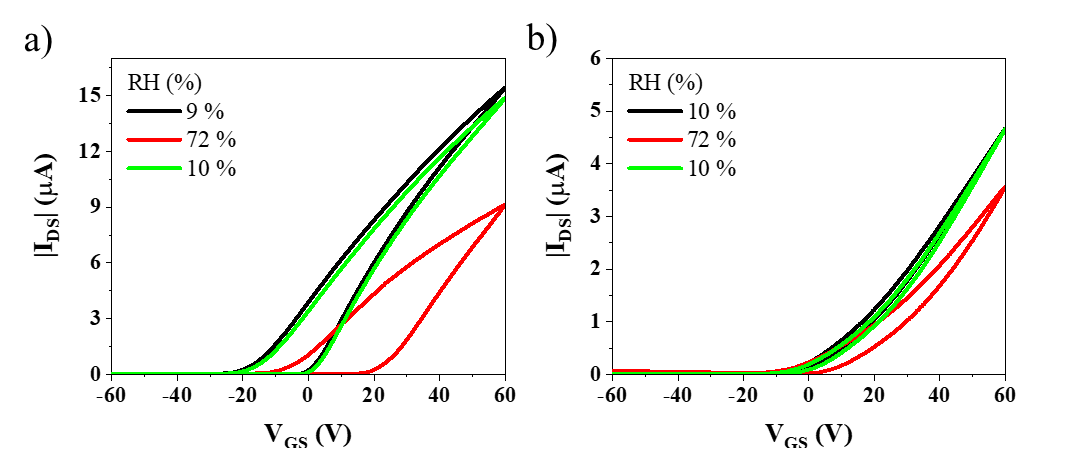


**Figure S17**. Transfer characteristics before exposure to high humidity, after reaching 72% RH, and after 1 hour of exposure to dry air to remove the absorbed water on (a) MoS_2_/SiO_x_, (b) MoS_2_/BCB/SiO_x_.


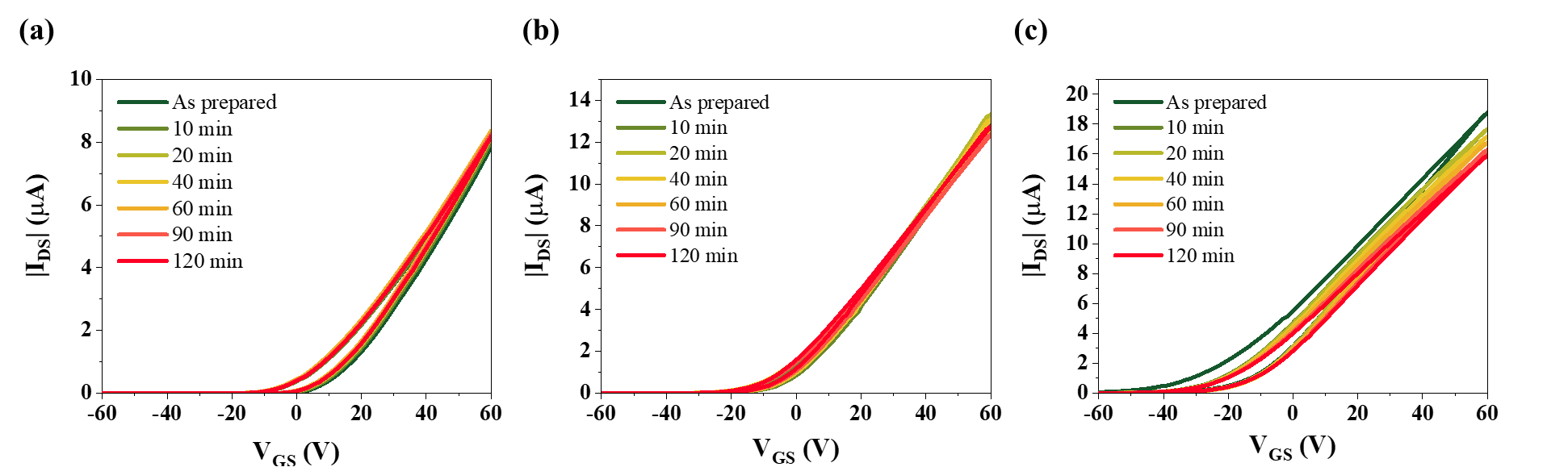


**Figure S18**. Transfer characteristics of (a) MoS_2_/SiO_x_, (b) EGO-SP/MoS_2_/SiO_x_ and (c) EGO-MC/MoS_2_/SiO_x_.
